# “I have travelled along on my own”—Experiences of seeking help for serious non‐COVID health problems during the COVID‐19 pandemic: A qualitative study

**DOI:** 10.1111/bjhp.12615

**Published:** 2022-07-26

**Authors:** Helen M. Parretti, Pippa Belderson, Helen Eborall, Felix Naughton, Yoon Loke, Nick Steel, Max Bachmann, Wendy Hardeman

**Affiliations:** ^1^ Norwich Medical School University of East Anglia Norwich UK; ^2^ School of Health Sciences University of East Anglia Norwich UK; ^3^ Usher Institute University of Edinburgh Edinburgh UK

**Keywords:** access, behaviour, COVID‐19, healthcare, help‐seeking, pandemic

## Abstract

**Objectives:**

During COVID‐19 the UK general population has been given strong messages to stay at home. Concurrently unprecedented changes occurred in healthcare access with moves to remote/triage systems. Data have shown that the number of people accessing healthcare services decreased and there are significant concerns that the pandemic has negatively affected help‐seeking for serious conditions, with potentially increased morbidity and mortality. An understanding of help‐seeking is urgently needed to inform public campaigns. We aimed to develop an in‐depth, theory‐based understanding of how, when and why people sought help for potentially serious symptoms (e.g., related to major cardiovascular events or cancer diagnoses) during the pandemic, and what influenced their decisions.

**Design:**

Qualitative semi‐structured interviews.

**Methods:**

We interviewed 25 adults recruited through a targeted social media campaign. Interviews were conducted via telephone or online platform. Our topic guide was informed by the Model of Pathways to Treatment and the Capability‐Opportunity‐Motivation‐Behaviour model.

**Results:**

The analysis identified four main themes: *Delay in recognition*, *Holding on to concerns*, *Weighing it up* and *Long‐term impacts*. Multiple societal and environmental factors influenced participants' help‐seeking and motivation, capability and opportunity to seek help, with long‐term impacts on well‐being and future help‐seeking.

**Conclusions:**

There is a need for clear guidance about pathways to raise concerns about symptoms and gain advice while usual healthcare contacts are paused or stopped. Recommendations for future interventions to support help‐seeking during pandemics include clearer messaging, co‐produced with end‐users, on when, where and how to seek help.

**Statement of contributions**

**What is already known?**

- The COVID‐19 pandemic led to significant changes in healthcare access and strong messages to “stay at home.”
- Data have shown that the number of people accessing healthcare services decreased.
- There are significant concerns that the pandemic has negatively affected help‐seeking for serious conditions.

**What does this study add?**

- A complex range of processes and influencing factors involved in seeking help for serious non‐COVID health problems.
- A key socio‐contextual factor was social disconnection impacting on recognition of abnormal symptoms and decisions.
- Need clear advice and reassurance on when, where and how to seek help for potentially serious conditions in pandemics.

## BACKGROUND

Since the first UK‐wide COVID‐19 lockdown (March 2020–July 2020), efforts have focused on infection control and clinical management of COVID‐19 (GOV.UK., 2020). However, concerns emerged that the pandemic response was negatively affecting conditions unrelated to COVID‐19 with 27.8% of the excess deaths between March and May 2020 not attributed to COVID‐19 (Office for National Statistics, 2020a). A total of 25,472 extra deaths occurred in private homes between December 2019 and September 2020 compared with the 5‐year average, with only 2358 due to COVID‐19 (Office for National Statistics, 2020b).

Healthcare service delivery in the United Kingdom changed significantly since the first national lockdown with reduced direct patient access and increased use of remote consultations (NHS Digital, 2020; The Health Foundation and Nuffield Trust, 2021). During the first lockdown, fewer people accessed the National Health Service (NHS) (i.e., 10% decrease in attended general practice [GP] appointments in March 2020 vs. March 2019 and a 49% decrease in Accident & Emergency attendances compared to pre‐lockdown) (Moynihan et al., 2021; NHS Digital, 2020; Public Health England, 2020a; Thornton, 2020). At the time this led to concerns that people were coming to harm by not seeking help, and the “Open for Business” campaign was launched by Public Health England (now Office for Health Inequalities and Disparities) (Bostock, 2020; Public Health England, 2020b; Thornton, 2020). Multiple studies have reported substantial reductions in emergency department attendances during the early stages of the pandemic (Reschen et al., 2021; Wyatt et al., 2021) and a systematic review of 81 studies across 20 countries found a median reduction in healthcare visits of 42% (interquartile range [IQR] −53% to −32%), a median reduction in admissions of 28% (IQR −40% to −17%) as well as reductions in diagnostics and therapeutics during the first lockdown (Moynihan et al., 2021). Of particular concern, hospital admissions have been reportedly reduced for conditions for which immediate care is crucial (e.g., myocardial infarction and appendicitis) (Blecker et al., 2021; Butt et al., 2020; Mafham et al., 2020; Oseran et al., 2020; Secco et al., 2020). Furthermore, cancer‐related admissions have shown a greater reduction than cardiovascular or respiratory‐related admissions (Shah et al., 2021).

The pandemic has also impacted the delivery of preventive healthcare (such as vaccinations and cancer screening) and non‐emergency surgical procedures, leading to concerns about the longer‐term adverse impact (Kursumovic et al., 2021; Maringe et al., 2020; McDonald et al., 2020). In summary, the literature suggests that fewer people were seeking help for all conditions—from acute and more life‐threatening to routine and preventive all of which could lead to potentially negative effects on health.

In terms of help‐seeking behaviours during the pandemic for non‐COVID‐19 health problems, in England, the GP patient survey (administered the first quarter of 2021) reported that 42% of patients who needed to see a GP in the previous 12 months had avoided making an appointment (NHS England, 2021). Similarly, a survey of people who had experienced a potential cancer symptom during the 6 months before September 2020 found that 44.8% had not contacted their GP (Quinn‐Scoggins et al., 2021). However, neither survey provided in‐depth insight into factors influencing people's decisions. To date, a small number of qualitative studies have explored help‐seeking during the COVID‐19 pandemic, but only included patients with specific health conditions (Ferry et al., 2021 (coronary heart disease); Steele et al., 2021 (eczema)).

Help‐seeking decisions depend on multiple factors, including those influenced by social and cultural contexts (Scott et al., 2013). How, when and why people decided to seek help for non‐COVID‐19 problems during the pandemic is not well understood. Given the ongoing changes to healthcare access and capacity issues (British Medical Association, 2021), seeking help for non‐COVID‐19 problems may become a long‐term problem, and a better understanding is needed to inform public health messaging. Two theoretical frameworks informed our study. Firstly, the Model of Pathways to Treatment (MPT) (Scott et al., 2013) which depicts the dynamic intervals of appraisal, help‐seeking, diagnostic interval and pre‐treatment, and describes processes within each interval and contributing factors. Some examples of contributing factors in the help‐seeking interval are previous experience and access to the healthcare provider. The use of the MPT helps identify problems that may be experienced along the help‐seeking pathway and potential target behaviours for future interventions on the care pathway. This includes appraising symptoms (e.g., shortness of breath attributed to COVID‐19 when actually a heart attack), and waiting to see if symptoms resolve before calling a GP and influences public health and media messages on decisions. Secondly, we used the Capability‐Opportunity‐Motivation‐Behaviour (COM‐B) model (Michie et al., 2011) to identify key modifiable influences (motivation, capability and opportunity) on important decisions and actions during this process, such as appraising symptoms and seeking help. Examples include people's knowledge about symptoms of a health problem (psychological capability), people's beliefs about the benefits of an early diagnosis (reflective motivation) to seek healthcare professional (HCP) advice for new symptoms, fear of contracting COVID‐19 in healthcare settings (automatic motivation), lack of social opportunity to discuss symptoms with friends and lack of physical opportunity to have a face‐to‐face consultation with HCPs.

We aimed to develop an in‐depth, theory‐based understanding of how, when and why people decide to seek help for acute or new potentially serious symptoms (e.g., related to major cardiovascular events or cancer diagnoses) during the COVID‐19 pandemic, to identify key modifiable influences on help‐seeking and taking action for future interventions.

## METHODS

### Study design

A qualitative interview study was conducted. Ethical approval was received from the University of East Anglia Faculty of Medicine and Health Sciences Research Ethics Committee on 10/2/2021 (reference: 2020/21–074).

The study was conducted in accordance with the Research Governance Framework for Health and Social Care, the applicable UK Statutory Instruments, (including the Data Protection Act 2018 [GOV.UK., 2018]) and the principles of Good Clinical Practice (Health Research Authority, 2020). Input on study design from 10 public contributors was included during the development of the study.

### Recruitment and sampling

We promoted the study on social media platforms, targeting patient organizations and professional networks. Examples included Facebook pages and Twitter accounts for Obesity UK, Healthwatch, Macmillan Cancer Support, Age UK, MIND, and Versus Arthritis. We “tagged” HCPs and academics with relevant expertise/speciality into Tweets, asking them to retweet, and asking administrators of patient/community Facebook forums, to share the post. Some organizations promoted the study via their web pages or mailing list. The University of East Anglia Health and Social Care Partnership and Faculty and Medical School advertised the study in newsletters.

The advertisement invited people who had experienced a serious non‐COVID health problem during the pandemic to take part in a study to hear people's experiences of seeking help for such problems (see Figure S1). Accompanying text included, “Have you been faced with a serious non‐COVID health problem during the pandemic? Where did you turn for advice and support? We'd like to listen to your experiences for our study” and a link to the online study invitation and participant information sheet. If interested, potential participants were asked to complete an online survey, which included demographics (age category, gender and postcode as a proxy of socioeconomic status), pre‐existing health conditions, carer status (have a formal/informal carer or any caring responsibilities), an outline of their help‐seeking experience and contact details. All responses were reviewed by PB. Eligibility included: ≥18 years and living in the United Kingdom, sufficient proficiency in English to participate in an interview and capacity to give informed consent. There were no eligibility criteria with regards to any specific underlying health conditions or symptoms for which help was sought, or additional exclusion criteria. We sampled purposively (within the pool of eligible respondents) to reach a range of participants in terms of demographics, a symptom experienced, pre‐existing health conditions and help‐seeking behaviour. Sample characteristics were reviewed at several points during data collection to inform decisions about further sampling. Participants sampled were telephoned or emailed by PB, who provided further information, checked willingness to participate and arranged a convenient time for an interview. Immediately prior to the interview, participants provided verbal consent (in addition to an emailed written consent form), which PB audio‐recorded separately. Recruitment ceased when we were satisfied that we reached saturation in responses to the specific research questions—in line with a *reflexive* thematic analysis approach, that is, at the point when distinctly new/different themes were arising in interviews, while acknowledging further fluidity in the emerging codes (Braun & Clarke, 2021).

### Data collection

We undertook in‐depth semi‐structured interviews, using a broadly narrative approach guided by a flexible topic guide. Interviews began with open questions about symptoms and circumstances when experiencing symptoms, with prompts, if needed, about any COVID‐19‐related restrictions impacting at the time. Subsequent questions probed decision making, help‐seeking and experiences of healthcare received.

The topic guide was informed by the MPT and COM‐B models (Michie et al., 2011; Scott et al., 2013) with questions included relating to the four intervals described by the MPT: appraisal (patient appraisal and self‐management), help‐seeking (decision to consult HCPs and arrange an appointment), diagnostic (HCP appraisal, referrals and appointments) and pre‐treatment (planning/scheduling of treatment); and to the patient, healthcare and disease factors that influence these intervals. The COM‐B model informed questions about influences on help‐seeking (such as motivation, social influences and physical opportunity). We included questions about wider socio‐contextual circumstances. We piloted the questions with clinical and academic colleagues and two acquaintances of the study team who had experienced seeking help during the pandemic and met the study criteria. Their feedback informed refinements to the topic guide and they provided advice on study advertising and recruitment.

Interviews were conducted by PB via a video conferencing platform or telephone, according to patient choice, aligning with our adherence to COVID‐19 restrictions at the time and commitment to participant safety. All were audio‐recorded (via platform's recording function or dictaphone). Interviews lasted 30–100 minutes (mean = 64) and were conducted between March and May 2021.

### Data analysis

Audio recordings were transcribed verbatim and anonymized. Our analytical approach was informed methodologically by Braun and Clark's thematic approach (Braun & Clarke, 2006), and drew theoretically upon MPT and COM‐B during the interpretation of the findings (facilitated by discussions with WH and FN). Data management was facilitated using qualitative data‐indexing software (QSR International NVivo version 12).

We undertook preliminary analysis on the initial nine interviews: PB read and produced descriptive analyses and summaries of each as single case studies; HP and HE read and coded two selected transcripts and we (PB, HP and HE) compared our coding (to assess consistency and agree with refinements) and discussed patterns to develop preliminary themes and the direction of further interviews. We held similar regular discussions throughout the data collection, reviewing data extracts in light of the preliminary themes and key research questions. This allowed emerging themes to be explored in later interviews and continued our approach to quality and consistency of coding; and also informed when to cease interviewing.

Following this initial inductive analysis, a broad coding frame was developed and agreed (PB, HP and HE), which PB used to analyse all transcripts, amending to add inductive codes as necessary. We shared emerging findings with the wider team mid‐way through analysis, aiding the development of interpretive themes.

## RESULTS

### Sample characteristics

Thirty‐seven people completed the survey; screening and sampling led to a final sample of 25 interview participants. Table 1 shows the sample demographic characteristics. The sample included a range of age groups under 70 years old. Participants reported a range of socioeconomic status, were mostly of White ethnicity and eight were male. Fourteen participants had a pre‐existing health condition, three had a formal carer and one had an informal carer. Three participants had caring responsibilities. Participants lived in a variety of geographical locations across England and one in Scotland.

**TABLE 1:** Sample characteristics

Characteristic	Frequency (N = 25)
Age group (years)	
18–30	4
31–40	4
41–50	5
51–60	7
61–70	5
Gender	
Male	8
Female	17
Ethnicity	
White	23
Black Caribbean	1
Mixed	1
Socio‐economic status	
Area (postcode) based Index of Multiple Deprivation Quintile (ascending SES)	
1 (most deprived)	4
2	9
3	1
4	4
5 (most affluent)	7
Had a carer	
Formal	3
Informal	1
None	21
Was a carer	
Yes	3
No	22
Pre‐existing long term condition	
Yes	14
No	11

Participants sought help for a variety of symptoms including severe pain, breathing problems, bleeding, lump and mental health symptoms such as anxiety and depression. Eventual diagnoses included significant conditions, some potentially life‐threatening, for example, pulmonary embolism, cancer, myocardial infarction and type 1 diabetes mellitus.

**FIGURE 1:**
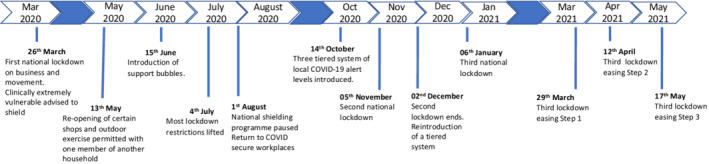
Timeline of COVID‐19 social distancing guidance in England, March 2020–May 2021 (Brown & Kirk‐Wade, 2021; Dunn et al., 2021)

Findings from the analysis of the transcripts were organized into four main themes: *Delay in recognition* (symptom attribution and influences on symptom recognition), *Holding on to concerns: delay in action* (attempts to self‐manage and influences that tended to delay help‐seeking), *Weighing it up and triggers to action* (multiple factors and influences considered in decisions to seek help) and *Long‐term impacts* (longer‐term effects on the individual of needing help for a potentially serious health problem during the pandemic). These main themes and the six sub‐themes within them are discussed below. A key cross‐cutting sub‐theme was the impact of social disconnection. Participant quotes are written in italic font with a participant ID number, age category and a broad category for the main diagnosis resulting from their symptoms given in brackets. Additional exemplar quotes are in Table S1. The timeline of social distancing guidance during the pandemic in England is summarized in Figure 1 to add context to the findings.

#### Delay in recognition

Participants described multiple factors relating to the pandemic that influenced their ability to recognize a bodily change or symptom, or to assess its severity as a precursor to seeking help. Some factors related to individual participants and their circumstances, while others related to the wider social context of a country experiencing a pandemic:”*my whole way I was living and using my body was changing”* [19F, 51–60, diagnosis awaited]. Some believed that prior to the pandemic, recognition, change and severity of their symptoms would have been far more apparent to themselves and others. Disruption to normal activities, working patterns and disconnection from social networks impacted symptom recognition in various ways, as described further in the below sub‐themes.

##### Social disconnection

Reduced face‐to‐face interaction within the workplace, and with friends and family, was reported to affect recognition. One man described how he felt like he “*was the last to recognise*” his deteriorating mental health, and that the enforced disconnection compounded this:

There was no way I could interact with anyone [working at home], so there's no way that anybody sort of knew what was happening and I was slowly sinking. [04 M, 61–70, mental health problem]

This could be particularly significant for those living alone and unable to see friends, family or partners who lived elsewhere. In contrast, some participants indicated that the easing of restrictions could help facilitate recognition:

I mean you know when [lockdown] was lifted and it was noticeable how ill I was and people did, family did comment that I wasn't very well. [08F, 51–60, venous thromboembolism]

One participant related how the introduction of social “bubbles” (see Figure 1), in which members of multiple households could mix, was a positive step in this respect, which allowed her to see her partner in person, facilitating recognition and subsequent help‐seeking.

##### Changes in life circumstances

Working at home featured as an influence on the recognition of healthcare needs, not only due to reduced social connection with colleagues but also because of the ergonomics of homeworking. One participant at first attributed her pain to working from home:

…I thought this [pain] is because I'm not sitting in a proper office chair. Life is very different. [21F, 51–60, cancer]

This participant also described trying to cope with symptoms while working at home, for example working on a laptop in bed to manage pain, in a way that would have been inconceivable had she been in the workplace.

Reduced activity due to lockdown measures also appeared to mask the extent of and recognition of symptoms. This was described by several participants whose leisure and routine activities (e.g., attending a gym or walking to work) were affected. This was particularly apparent for symptoms such as chronic pain or breathing problems.

When you're at home just sitting around it's not that noticeable […] it's not making such a difference. […] I would have realised that I couldn't walk properly, whereas I didn't have to go out. [08F, 51–60, venous thromboembolism]

This effect could be magnified for those instructed to “shield” (people deemed clinically extremely vulnerable were advised to “shield” [remain at home and have no contact outside of the house], see Figure 1); the sudden shift to being entirely confined to the home could result in a lack of being attuned to the full impact of symptoms on normal functioning:

…because I wasn't going anywhere anyway, it didn't really matter if I couldn't breathe, whereas if I was like going into work and things like that, it would have been much more of an issue. [24F, 18–30, airway problem]

Conversely, the effects of taking more exercise could also impact recognition of the issue—one participant had seized the opportunity to go for the permitted daily hour's walk and at first thought this was the reason for her aching limbs (cancer diagnosis).

Changes to diet in response to lockdown were also raised as contributing to delayed recognition, for example, one participant related how at first she thought her symptoms during the first lockdown were due to drinking more alcohol and eating less healthy foods.

Some participants also reported attempts at self‐diagnosis. Sometimes incorrectly, delaying the recognition of symptoms requiring help, sometimes over many months. Examples of self‐diagnosis in which symptoms were misinterpreted included significant conditions such as pulmonary embolism mistaken as asthma, type 1 diabetes mistaken as stomach upset and stress. This could also apply to mental health symptoms, for example, one woman described how she initially attributed her symptoms as a reaction to the stress she felt when entering the first lockdown and her husband's instruction to shield her:

I put it down to sort of being low level anxiety […] I noticed the difference in bowel habits, but then we were in extraordinary situation […]. So it didn't seem unreasonable to me that something would be affected by that. [20F, 61–70, cancer and lymphocytic colitis]

#### Holding on to concerns: Delay in action

A recurrent thread through many accounts was the sense of *“holding on to concerns”* in a way that they would not have done before the pandemic. For some, this was part of a broader sense of *“life on hold”* precipitated by the pandemic (*“everything just felt so paralysed”* [07F, 31–40, cancer]) and becoming accustomed to waiting, for a return to normality or for the pandemic to be over. This mentality of the retreat was sometimes interlaced with incomplete recognition, and a hope that symptoms would naturally resolve:

I suppose that sort of set the tone for the year really […]. You just kind of gave up [seeking help] and really there was this kind of sense that you were on your own […] I think people have sort of shut down rather a lot in the pandemic […] haven't wanted to sort of cause a fuss, maybe even talk about their health because we all know there's this huge thing going on. [19F, 51–60, diagnosis awaited]

For others, the sense of holding on to concerns manifested in accounts of self‐assessment and self‐management and in navigating decisions in light of the risk of exposure to COVID‐19.

##### Self‐assessment and self‐management

Many participants spoke about how the pandemic had instigated greater attempts at self‐reliance, as one participant put it: *“Trying to be your own doctor”* [06F, 41–50, diagnosis awaited]. Many examples were mentioned of self‐management of conditions, including altering diet and lifestyle, using or adjusting doses of medications (e.g., painkillers and indigestion remedy), or purchasing specialist equipment.

Take some Ibuprofen when it bothered me. Try to break up my walk […] I bought some proper walking shoes, in that time as well, just to try to self‐medicate really. [02F, 31–40, ophthalmological and joint problems]

Many participants described judgement calls about when symptoms merited attention. Where symptoms felt severe or constituting an emergency, this could facilitate a quick judgement that this required immediate attention. Many felt that the pandemic had raised the threshold for taking this decision, sometimes despite experiencing symptoms that were hard to bear:

I mean it was daft now I look back on it I could hardly breathe I was sticking my head out the window all the time on the landing trying to get some air in, it was really bad, but I just didn't think I was ill enough. [08F, 51–60, venous thromboembolism]

##### Risk of exposure to COVID‐19

While the risk of exposure to COVID‐19 did not feature as a barrier to seeking help for some, for others anxiety about contracting COVID‐19 by attending healthcare settings reportedly played a role in their decision‐making, especially in the early stages of the pandemic:

Even though the symptoms were gradually getting worse and more frequent, I didn't actually want to phone up. Because it was in me head ‘What happens if they say yes, come into hospital?’ I've got to go in a hospital and might end up getting COVID. So I was battling with my own insecurities, demons, regarding COVID‐19 and not wanting to seek treatment but at the same time battling in my head as to what's wrong with me? […] I was stuck between a rock and a hard place as to asking for help. [06F, 41–50, diagnosis awaited]

The risk of exposure to COVID‐19 was a particular concern for those who had been identified as clinically extremely vulnerable. One woman described how receiving her instruction to shield:

kind of panic set in, because then you're like, who do you go to for help? So it's very stressful. And then on the news you could hear about how bad it was in the hospitals. So then you're kind of stuck. [25F, 41–50, diagnosis awaited]

This could result in a paradoxical effect of shielding, which although intended to protect the health, promoted a sense of isolation and fear about seeking help from health services:

When you're constantly being told that you shouldn't reach out to anyone because that's unsafe, yeah it kind of reinforces this idea of like – ‘OK well I just won’t seek help, I won't see anyone, I won't do anything, I'll just stay here in my bubble’. [24F, 18–30, airway problem]

Some also described a hesitancy borne of altruistic concerns about passing the virus to healthcare workers.

##### Concerns about burdening healthcare services

A sense of duty not to burden services during the pandemic was a predominant theme across interviews.

It was almost like, ‘oh, I don't want to bother them, I don't want to disturb them’. I was picking up was that COVID was causing a lot of extra work for the NHS and I guess I was thinking ‘gosh that’s really bad they've got lots of work already they don't need any more’. [08F, 51–60, venous thromboembolism]

Related to this sense of duty was a perception that COVID‐19 was the priority and that others were in greater need, as illustrated by a participant who ultimately required emergency admission:

going into hospital and getting treatment is selfish because you haven't got COVID and there's people who have who need the care more. [22 M, 18–30, endocrine problem]

Not wanting to burden services impacted the choice of contact when deciding to seek help; one participant phoned her GP rather than 999 because of concerns about pressure on the ambulance service; another consulted a private GP to validate subsequently making an appointment with an NHS doctor. Hesitant about how to proceed, several participants phoned NHS 111 to validate their decision (NHS 111 is a helpline in the United Kingdom for the general public to call and gain advice for symptoms. Appointments can be arranged for the person to see a GP, advice given to attend A&E or an ambulance called, as appropriate):

I suspected that they didn't want people turning up who you know, unless you were really in need. And kind of ringing 111 validated my decision to go […] if you get told to go to A&E then you feel kind of better about it. [14M, 51–60, ophthalmological problem]

Messaging from media, government or healthcare services at the pandemic's outset had set the tone for this sense of duty. Media reports and images of busy hospital wards, particularly the early stark images of overwhelmed hospitals, added to fears that health services were overwhelmed and help was not accessible.

Everyone was dissuaded from contacting your doctor or any medical profession, and because of COVID […] unless you were dying in the street and […] because they were so busy dealing with COVID, and with the constant TV ads, you were constantly being reminded. [21F, 51–60, cancer]

The expectation of delays, unavailable or limited services, could serve to reinforce the strategy of holding on to concerns:

They've got enough going on or there's no point in phoning up because they won't be there. Everybody is dealing with COVID‐19. [06F, 41–50, diagnosis awaited]

Some commented that public health campaigns aimed at encouraging people to seek help if needed did not always reflect their experiences in reality:

You'll get a national message that will go ‘oh, we're all worried that people aren't consulting’ but when you actually try, it's really hard because they're still not really open. [19F, 51–60, diagnosis awaited]

For some, the sense of confusion about what to expect from services due to COVID‐19 could contribute to a negative feedback loop, whereby they felt alone and continued to hold on to concerns. Interviews demonstrated the importance of good communication in the context of isolation and uncertainty.

I think perhaps more positive messages about how things are working, clearer information […] I don't think any of us mind waiting for these things if you know why you're waiting […] it is having a clearer picture of why. [01F, 61–70, hepatobiliary problem]

The loss of routine healthcare and review appointments also reduced opportunities to communicate concerns to an HCP or, indeed, for HCPs to notice concerning symptoms themselves.

They cancelled a lot of reviews, I didn't have my normal thyroid review blood tests […] I may have [mentioned gastro symptoms then], it is only usually a blood test but I do sometimes mention and quite often they will just say well you need to make an appointment so it probably would have prompted, I think, if I'd been able to go for that. [01F, 61–70, hepatobiliary problem]

Some described actively exploring alternative sources of help because of the pandemic, for example, taking part in online support groups, rather than seeking help from an HCP.

#### Weighing it up and triggers action

**FIGURE 2:**
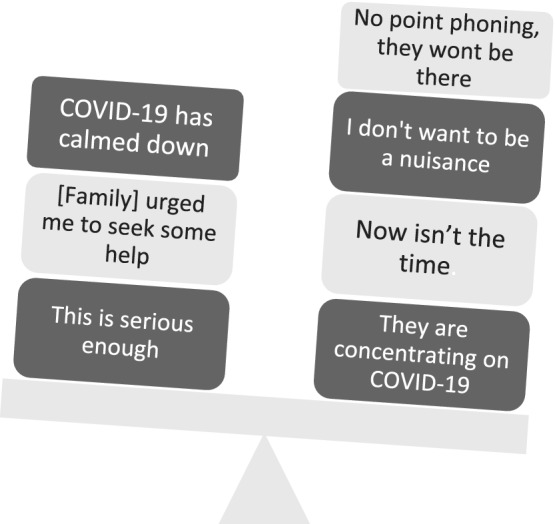
“Weighing it up”

Participants described a process of *“weighing‐up”* when to seek help as well as deciding on the extent and nature of help to seek. Multiple factors influenced this, including those described above as well as the type and perceived urgency of symptoms experienced. However, the pandemic tended to add weight against seeking help, delaying action (Figure 2).

Balancing up in your mind, thinking is this something that's worth me bothering the doctor about? [21F, 51–60, cancer]

Many described a catalyst, which tipped the balance towards triggering action and seeking help. The most common catalyst was a recognition that symptoms were “bad enough” or worsening. Other catalysts included: reaching a point of struggling to cope, re‐evaluating self‐diagnosis, changing perceptions of NHS capacity and changes to media or service messaging, including changes to the guidance on social contacts.

The number of cases was not so high at that point, it was really low. So that also […] played a role because I thought, okay the risk now is generally not high […] once the risk of potential threat of the problem was greater than the threat of the pandemic, I didn't hesitate to access healthcare at all. [16F, 31–40, throat problem]

By [August] I felt everyone is getting on with their life and I shouldn't be so worried about putting pressure on the NHS. [01F, 61–70, hepatobiliary problem]

Prompting by others, including family and friends, HCPs or a helpline, was a notable catalyst; for example, one man, who was hospitalized with deep vein thrombosis, credited his wife's influence on his decision to seek help:

Having my wife kind of there to say, ‘well, I don't like this ‐ please do something about it’ was again another factor in going to 111 when I did. So I can't say for sure, but I probably would have waited a couple more days before I did if it weren't for that. [05 M, 31–40, venous thromboembolism]

A further catalyst was the opportunity for conversation with others such as work colleagues. One participant described how a daily [Microsoft] Teams call with her team, which incorporated informal conversation, was instrumental to her decision to seek help:

[a colleague] shared their experience [of attending a health appointment] and, me being scared about it, I was asking how did you find it? And once they'd shared how well organised it was […] I suppose that also gave me confidence then. [16F, 31–40, throat problem]

However, uncertainty and heightened confusion about the appropriate NHS port of call once a decision to seek help was made was apparent:

You didn't know exactly where to go to, if you went to the GP or whether to call 111 […] I think the pharmacist ended up taking a lot of the brunt of it […] there was a communication vacuum […] So you were kind of left and you kind of felt abandoned. You didn't have a clue what to do, where to go, who to talk to. You didn't have your normal appointment, so then you didn't know what was happening with that. Or if you could contact the hospital as well, so it was kind of a no man's land… [25F, 41–50, diagnosis awaited]

##### Assets

Help‐seeking behaviour was also underpinned by access to assets, whether this was social capital in the form of connection to health expertise via personal or professional networks, access to and familiarity with online technology to enable help‐seeking, access to private car or the ability to afford taxis to attend appointments (in the context of fear about the risk associated with using public transport), or knowledge, as in the case of several participants who worked in the healthcare sector and felt more capable to take quick and informed decisions about accessing help. Notably having the financial means to access private care was apparent. Several participants had turned to the private sector specifically because of the pandemic, anticipating delay; to gain diagnosis and treatment or to validate their decision to approach an NHS doctor.

but if it did show a cancer, I would have booked […] privately as well. Within a couple of days privately rather than the NHS […]. Yeah, how I access services going forward I'll probably use more private services. [3 M, 18–30, urological problem]

#### Long‐term impacts

The experience of needing to seek help for a serious health problem during the pandemic adversely impacted many participants' mental health. This was related to navigating uncertainty, being diagnosed with a new condition which sometimes also elevated their clinical vulnerability to COVID‐19, guilt for “burdening” services, regret or self‐blame for not seeking help at an earlier stage, and exacerbation of existing mental health problems:

It's just magnified everything that I was already struggling with. [06F, 41–50, diagnosis awaited]

Coping alone was a striking and recurrent thread throughout the interviews.

“I have travelled along on my own” **[**07F, 31–40, cancer**]**

There were varying degrees of this from “fending for myself” but coping well, to feeling completely abandoned. For some, there was an acute sense of having been cast adrift, abandoned and forgotten. This was described as a product of both enforced isolation and the efforts to deal with and prioritize COVID‐19:

It does feel like you're sort of forgotten in the need to look after the COVID side of things. [01F, 61–70, hepatobiliary problem]

Enduring behavioural change was also evident, with continued concern about adding to waiting lists and backlogs. One woman, diagnosed with cancer, described continuing hesitance to seek help, over a year from the start of the pandemic, despite the gravity of her diagnosis:

I'm still doing it and you know I've got a horrendous prognosis and disease, and I'm still thinking I'm not sure I should keep on contacting the doctor about anything […] there was such an emphasis on COVID you were made to feel guilty for trying to access any kind of help. And I still feel that's the case and I'm still worried about picking up the phone to trying to get an appointment to see my GP because the waiting lists are huge. [21F, 51–60, cancer]

However, there was also a demonstration of agency in the face of changes and challenges experienced, as one participant put it: “*I'm finding my own way through”* [07F, 31–40, cancer]. Adaptive strategies had been adopted in response to changes experienced, for example, visiting pharmacies at quieter times, finding alternative sources of support (e.g., online groups) or ways to make remote appointments workable (e.g., making notes during appointments). There were also accounts of resilience and enhanced confidence:

I have to say I'd probably rely on my own resources as much as possible […] I do think there's a bit of a change around that. I don't think it's just me, when I've talked to other people, I think that there is much more reliance on trying to sort yourself out, which might be a good thing long term. [11F, 41–50, cardiac problem]

## DISCUSSION

Our findings have revealed the range and complexity of the processes and influencing factors involved in seeking help for serious non‐COVID‐19 health problems during the pandemic. Interviews were informed by the MPT and our themes generally aligned well with the intervals described in the MPT (Scott et al., 2013). For example, *Delay in recognition* describes similar processes to those within the Appraisal interval, while *Holding on to concerns: delay in action*, *Weighing it up and triggers to action* are aligned with the processes described in the help‐seeking interval. However, our findings extended beyond this model and include processes and influences related to accessing and receiving help as well as longer‐term impacts of help‐seeking experiences. The pandemic restrictions significantly changed wider socio‐contextual factors: social disconnection, changes to life circumstances and wider societal, environmental and individual‐level factors, as discussed below. These are not accounted for by the MPT but influence capability, motivation and opportunity as behavioural determinants defined by the COM‐B model (Michie et al., 2011). For instance, the lockdown reduced participants' physical and social opportunities to appraise symptoms and seek help due to social distancing and reduced healthcare access. It also reduced their reflective motivation to seek help due to the public health messaging due to an overburdened NHS and the risk of contracting COVID. Anxiety‐provoking media images may have reduced their automatic motivation to seek help. Finally, the lockdown reduced participants' psychological capability to appraise symptoms and seek help due to a lack of perceived and actual social support; and reduced their physical opportunity to seek help and access the NHS through the loss of routine appointments.

Social disconnection was a key socio‐contextual factor and a sub‐theme that ran through our findings, which had a significant impact on opportunities to recognize bodily changes as the abnormal, capability to assess their severity and decisions to seek help. The impact of isolation and the importance of social networks in encouraging help‐seeking have been described in pre‐COVID‐19 studies, as well as in a study exploring experiences of chronic pain during COVID‐19 (Amja et al., 2021; Hall et al., 2015; Walter et al., 2014). In our study, the considerable reduction in social contacts during COVID‐19 seems to have led to a significant loss of these opportunities to seek help, which was further magnified for those shielded and deemed clinically extremely vulnerable. Indeed, evidence suggests that mortality in the clinically extremely vulnerable population was >2.5 times that of the general population at the peak of the first COVID‐19 wave and that they were particularly affected by the changes to NHS services during the pandemic (Hodgson et al., 2021).

Another key socio‐contextual factor was changes in life circumstances during the pandemic, which reduced people's opportunities and capability to recognize symptoms. Generally, these changes led to a delay in recognition and seeking help, consistent with a previous study, which found that people were more likely to recognize a bodily change as a symptom if it interfered with daily activities, and to seek help when the symptom became a threat to normal life (Hall et al., 2015). However, the substantial disruptions to daily activities due to COVID‐19 led to “normal life” becoming less clear and reduced opportunity for symptom recognition.

During the early phases of the pandemic, social norms were significantly disrupted by the rapid development of new norms (such as social distancing), contributing to people's uncertainties about when to seek help and reducing social opportunity to seek help (Levealahti et al., 2007). In addition, the altered landscape of NHS healthcare services reduced physical opportunity to seek help and increased people's feelings of uncertainty, leaving them confused when trying to find appropriate routes to care. Other societal factors also influenced participants' beliefs about their illness and symptoms, such as identity, timeline, cause, control/cure and consequences (Leventhal et al., 1980).

Communications and messaging from healthcare services, the government and the media were also important societal influences. Messaging particularly influenced decision‐making to take action following symptom recognition and appears to have led to a form of cognitive dissonance (Festinger, 1957) with people feeling increasing concern about their symptoms while also feeling concerned or fear about seeking help and the consequences of taking action. This appeared to result in the endorsement of “disengagement beliefs” (Bandura et al., 1996) that may relieve the psychological discomfort generated by the dissonance. These beliefs included “I don't want to be burden to the NHS” and “there's no point in phoning as HCPs won't be there” (beliefs around “not being a burden” were also voiced in the study by Quinn‐Scoggins et al. (2021) and the GP patient survey (NHS England, 2021)). Indeed, participants in our study described using NHS 111 to legitimize seeking help, which is reflected in national statistics showing increased use of NHS 111 during the pandemic (Healthwatch, 2021). Previous studies on help‐seeking have described similar needs to legitimize use of healthcare services and not to waste doctor's time (Hall et al., 2015; Walter et al., 2014).

### Strengths and limitations

This study's strengths include reaching a diverse sample in terms of health problems experienced, gender, socio‐economic status and geographical area. In addition, the topic guide was informed by theoretical frameworks, clinicians and patients. Limitations of the study were associated with recruiting via social media and patient organizations; it is likely we did not reach patients with limited access to digital technology, including older adults, and our sample was predominantly White British. Future research needs to reach underserved communities, including those from a range of ethnicities and will require more targeted recruitment methods to effectively reach people from within those communities. It would be particularly important to hear from people from diverse ethnic groups given there is some evidence that reductions in hospital admissions during the first year of the pandemic were higher in those of Mixed, Other or Black ethnicity than in those of White ethnicity (Shah et al., 2021). Our study was conducted in early 2021, about a year after the pandemic began, and therefore there is the potential for recall bias and post‐hoc rationalization of experiences.

This study focussed on the experiences of patients seeking help for themselves whereas future research could also explore the experiences of family and carers accessing help for others. Exploring the experiences of HCPs working in front‐line settings would also further our understanding of seeking and receiving help during a pandemic.

There has been some criticism in the literature around the use of the word “delay,” for example in studies of patients with cancer (Dobson et al., 2014). However, conceptually “delay” was felt to best reflect the range of narratives participants shared in this study, which varied from passive inertia to purposeful postponement as a product of external restrictions and context.

### Implications and recommendations

When translating the findings into recommendations for interventions to facilitate appropriate decision‐making and action around help‐seeking, we drew upon MPT to interrogate our analyses relating to the four intervals of help‐seeking; and the COM‐B model to consider the different influences on help‐seeking. The wide range of influences on appraisal and help‐seeking behaviours, in turn, influenced by wider‐level factors, highlights that public health campaigns need to consider and target all these modifiable influences and factors when encouraging people to seek help for serious non‐COVID related problems.

When drawing up policy and advice about accessing healthcare in future pandemics (and similar situations), policymakers would benefit from recognizing the challenges for individuals in assessing and self‐managing new and uncertain symptoms, that even highly experienced clinicians struggle with. Any firm “stay at home” message, needs to be accompanied by safeguards for enabling easy access to trusted advice so that people can recognize when leaving home is the officially recommended option without guilt or fear. Indeed, although simple and clear messages are required during a pandemic, they also need to include clear advice on when, where and how to seek help for potentially serious conditions (i.e., action planning) and reassure that this is socially acceptable.

In addition, clear advice must be provided about pathways for raising concerns about symptoms and seeking advice while routine healthcare services are paused or stopped during a dynamic situation. Similarly, clear advice must be provided regarding what to expect when attending a healthcare service during a pandemic to reassure patients to seek help when needed and increase psychological capability.

Public health messaging could encourage people to regularly check on vulnerable friends/family during a pandemic to increase opportunities for symptom recognition and appraisal. Co‐production of campaigns and messages with key stakeholders is crucial to ensure that messages are clear, simple, and enactable.

In the medium to long term, and as services return to pre‐pandemic functioning, people's experiences of help‐seeking and decision‐making in the context of pandemic restrictions will continue to have long‐term impacts, for example, on mental health and future help‐seeking behaviour. It is important that HCPs are mindful of this during interactions with patients.

## CONCLUSIONS

The COVID‐19 pandemic has had a substantial impact on the delivery of and access to care within the NHS. A major part of this burden fell on patients who needed to develop sophisticated self‐management skills in judging the seriousness and priority of their health problems. This was exacerbated by changes in access to healthcare and surges of COVID‐19‐related hospital admissions adding to patient anxieties.

The pandemic was found to influence all stages of help‐seeking. Concerningly, some report changes in their help‐seeking behaviour persisting even after a diagnosis of a serious health condition. Multiple wider societal and environmental factors influenced motivation, capability and opportunity to appraise symptoms, seek help and access services, which tended to result in delayed decisions. Key factors specific to the pandemic were social disconnection, changes in life circumstances and messaging. Media, government, public health and healthcare service messaging could contribute to a delay in seeking help and/or uncertainty about appropriate pathways to help.

Our study has significantly contributed to an understanding of how, when and why people sought help for serious non‐COVID health problems during the pandemic and identified key influences on those decisions and actions. Study findings have allowed preliminary recommendations for policymakers, public health and healthcare organizations to be suggested, informed by behavioural theory.

## AUTHOR CONTRIBUTIONS

**Helen M Parretti:** Conceptualization; data curation; formal analysis; funding acquisition; writing – original draft; writing – review and editing. **Pippa Belderson:** Data curation; formal analysis; project administration; writing – original draft; writing – review and editing. **Helen Eborall:** Conceptualization; formal analysis; funding acquisition; methodology; writing – original draft; writing – review and editing. **Felix Naughton:** Conceptualization; formal analysis; funding acquisition; methodology; writing – review and editing. **Yoon Loke:** Conceptualization; formal analysis; funding acquisition; writing – review and editing. **Nick Steel:** Conceptualization; formal analysis; funding acquisition; writing – review and editing. **Max Bachmann:** Conceptualization; formal analysis; funding acquisition; writing – review and editing. **Wendy Hardeman:** Conceptualization; formal analysis; funding acquisition; methodology; writing – original draft; writing – review and editing.

## FUNDING INFORMATION

This study was funded by University of East Anglia (UEA) Health and Social Care Partners.

## CONFLICT OF INTEREST

All authors declare no conflict of interest.

## INFORMED CONSENT TO PARTICIPATE

All participants provided verbal or written informed consent to participate in all aspects of this research.

## Supporting information


Appendix S1

## Data Availability

The datasets used and analysed during this study are available from the corresponding author on reasonable request. Access to anonymized data may be granted following a review of the request. Exclusive use will be retained until the publication of major outputs from this research.
