# Supplementary material for: “I have travelled along on my own”—Experiences of seeking help for serious non‐COVID health problems during the COVID‐19 pandemic: A qualitative study
Source: Br J Health Psychol. 2022 Jul 26:10.1111/bjhp.12615. Online ahead of print. doi: 10.1111/bjhp.12615 (PMC9349934; doi:10.1111/bjhp.12615)
Supplement: Supplementary file 1 — Appendix S1 [file BJHP-9999-0-s001.docx]

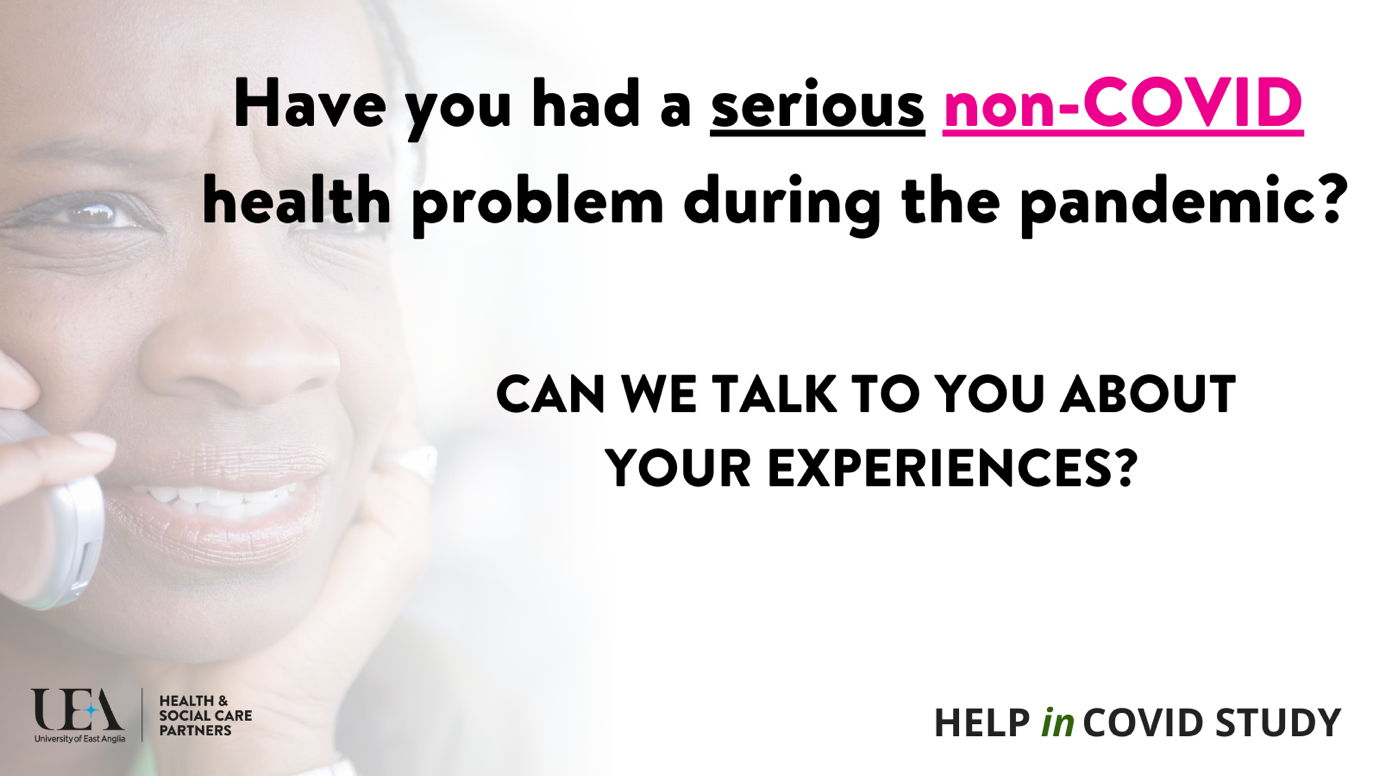


**Figure S1:** Example recruitment advert (Twitter)

| **Theme/sub-theme** | **Quote** |
| --- | --- |
| **Holding on to concerns: delay to action**  ***Self-assessment and self-management*** | *“I thought it was an emergency so that sort of trumped everything”* [10F, 51-60, ear and gynaecological problems].  *“I sort of didn't reach out initially because I was hoping that it would just sort of resolve itself and because I wasn't really going out much or doing or being physically active, so I felt like it was manageable”* [24F, 18-30, airway problem].  *“It sounds a bit pathetic looking back at it. I just thought if I have two paracetamol it might all just go, you know”* [14M, 51-60, ophthalmological problem]. |
| **Holding on to concerns: delay to action**  ***Concerns about burdening healthcare services*** | *“The reason I phoned 111 rather than taking myself off to A&E when I was…sort of thinking ‘Well, I'll check someone else’s opinion before I make a nuisance of myself here’…I think it definitely is worse for the pandemic …”* [10F, 51-60, ear and gynaecological problems].  *“You saw it on the TV about how busy the hospitals are…I thought the last thing they need is me turning up that might be something and nothing, and bothering them”* [14M, 51-60, ophthalmological problem]*.*  *“…it’s a lot easier to get a GP’s appointment when I’m at home all day and I can speak to the GP over skype or whatever…I think it made my diagnosis, I got my diagnosis earlier because I wouldn’t have been able to fit in a GP appointment”* [07F, 31-40, cancer].  *“I was struggling to see my GP for a review of that so I thought [symptoms were] because I didn’t have my medication for that”* [22M, 18-30, endocrine problem].  *“I’ve started taking part in a coffee group which is to help people struggling with mental issues, just to have a place to talk […] Just to help with mental illness. We don’t want to overwhelm the medical profession, they’re going through enough, so I’m trying to do my bit and keep my sanity at the same time”* [23F, 51-60, mental health and neurological problems]. |
| **Weighing it up: triggers to action** | *“I wasn't able to spend time with my boyfriend 'cause he's not a part of my household […] if he was spending time with me, more like normal, he would have said like, ‘This sounds bad, you should probably see someone about this’” [24F, 18-30, airway problem].* |
| **Long-term impacts** | *“It's like there's only one lot of people who matter in this. We don’t matter”* [13M, 61-70, neurological problem].  *“It’s been made harder by the pandemic, because I think people can't see me as much and they can't see perhaps the impact it's having on me, so I've really noticed that. It's felt a bit lonely actually […] felt a bit left to get on things from my GP and I feel, yeah, perhaps I haven't got some of the support I might have had from friends and family” [10F, 51-60, ear and gynaecological problems].*  *“I got around it by making sure that I made notes. Where [previously] I wouldn’t normally be doing that, I’d rely on whoever came with me to sort of remember stuff I didn’t” [17F, 18-30, respiratory problem].* |

**Table S1:** Additional exemplar quotes
